# PEP-1–PIN1 Promotes Hippocampal Neuronal Cell Survival by Inhibiting Cellular ROS and MAPK Phosphorylation

**DOI:** 10.3390/biomedicines12102352

**Published:** 2024-10-15

**Authors:** Jung Hwan Park, Min Jea Shin, Gi Soo Youn, Hyeon Ji Yeo, Eun Ji Yeo, Hyun Jung Kwon, Lee Re Lee, Na Yeon Kim, Su Yeon Kwon, Su Min Kim, Yong-Jun Cho, Sung Ho Lee, Hyo Young Jung, Dae Won Kim, Won Sik Eum, Soo Young Choi

**Affiliations:** 1 Department of Biomedical Science and Research Institute of Bioscience & Biotechnology, Hallym University, Chuncheon 24252, Republic of Korea; hwanee15@hallym.ac.kr (J.H.P.); wehome3@hallym.ac.kr (M.J.S.); gsyoun@hallym.ac.kr (G.S.Y.); hjyeo@hallym.ac.kr (H.J.Y.); ejyeo@hallym.ac.kr (E.J.Y.); donuts25@hallym.ac.kr (H.J.K.); 44225@hallym.ac.kr (L.R.L.); 44310@hallym.ac.kr (N.Y.K.); 44309@hallym.ac.kr (S.Y.K.); ksm16@hallym.ac.kr (S.M.K.); iamsungho@gmail.com (S.H.L.); 2 Department of Neurosurgery, Hallym University Medical Center, Chuncheon 24253, Republic of Korea; nssur771@hallym.or.kr; 3 Genesen Inc., Teheran-ro, Gangnam-gu, Seoul 06181, Republic of Korea; 4 Department of Veterinary Medicine & Institute of Veterinary Science, Chungnam National University, Daejeon 34134, Republic of Korea; hyjung@cnu.ac.kr; 5 Department of Biochemistry and Molecular Biology, Research Institute of Oral Sciences, College of Dentistry, Gangneung-Wonju National University, Gangneung 25457, Republic of Korea; kimdw@gwnu.ac.kr

**Keywords:** ischemia, PEP-1–PIN1, oxidative stress, MAPK, protein therapy

## Abstract

**Background***:* The peptidyl-prolyl isomerase (PIN1) plays a vital role in cellular processes, including intracellular signaling and apoptosis. While oxidative stress is considered one of the primary mechanisms of pathogenesis in brain ischemic injury, the precise function of PIN1 in this disease remains to be elucidated. **Objective***:* We constructed a cell-permeable PEP-1–PIN1 fusion protein and investigated PIN1’s function in HT-22 hippocampal cells as well as in a brain ischemic injury gerbil model. **Methods***:* Transduction of PEP-1–PIN1 into HT-22 cells and signaling pathways were determined by Western blot analysis. Intracellular reactive oxygen species (ROS) production and DNA damage was confirmed by DCF-DA and TUNEL staining. Cell viability was determined by MTT assay. Protective effects of PEP-1-PIN1 against ischemic injury were examined using immunohistochemistry. **Results**: PEP-1–PIN1, when transduced into HT-22 hippocampal cells, inhibited cell death in H_2_O_2_-treated cells and markedly reduced DNA fragmentation and ROS production. This fusion protein also reduced phosphorylation of mitogen-activated protein kinase (MAPK) and modulated expression levels of apoptosis-signaling proteins in HT-22 cells. Furthermore, PEP-1–PIN1 was distributed in gerbil hippocampus neuronal cells after passing through the blood–brain barrier (BBB) and significantly protected against neuronal cell death and also decreased activation of microglia and astrocytes in an ischemic injury gerbil model. **Conclusions***:* These results indicate that PEP-1–PIN1 can inhibit ischemic brain injury by reducing cellular ROS levels and regulating MAPK and apoptosis-signaling pathways, suggesting that PIN1 plays a protective role in H_2_O_2_-treated HT-22 cells and ischemic injury gerbil model.

## 1. Introduction

Peptidyl-prolyl isomerase (PIN1) is primarily found in the nucleus of neurons and plays a crucial role in various cellular processes, including aging and neurodegenerative disorders. In addition, it regulates multiple physiological processes, such as the cell cycle, proliferation, differentiation, cell death, and DNA damage and repair [1,2,3,4,5]. Several studies have shown that PIN1 activity is decreased in the hippocampus of Alzheimer’s disease (AD) patients, which means that this protein plays important roles in the neuronal cell death pathway, in turn suggesting that PIN1 has a neuroprotective role in AD [3,6,7]. It has also been reported that a loss of PIN1 induces senescence and that PIN1 knock-out mice show a senescent phenotype [8,9]. Additionally, PIN1 expression in the myocardium is decreased with aging, suggesting that PIN1 is an important anti-aging protein [10]. Though PIN1 involves the various cellular process, including brain diseases, the role of this protein in ischemic injury has not yet been investigated.

It is known that transient forebrain ischemia is caused by an insufficient blood supply to the brain and that this insufficient blood supply affects oxygen and glucose concentrations in neurons and glia, ultimately causing neuronal cell death in the brain [11,12], and that the mechanism of this cell death is linked to oxidative stress, excitotoxicity, and inflammatory responses [13,14]. Many reports have shown that ischemic injury can markedly increase ROS levels and induce oxidative damage in neurons [15,16] and that this ROS-induced oxidative stress plays a crucial role in neuronal damage in the hippocampus [17,18,19].

It has been reported that excessive production of ROS is associated with neuronal disorders of apoptosis signaling [20,21,22,23] and that mitogen-activated protein kinase (MAPK) is involved in cell survival and apoptosis [24,25,26]. Therefore, studies of the modulation of ROS and MAPK signaling pathways that aim to protect neuronal cells against damage need to be elucidated. To attempt to deliver the therapeutic molecules into the brain for the prevention or reduction of brain damage after an ischemic injury, passage through the blood–brain barrier (BBB) is the most important factor. The protein transduction domain (PTD) can solve this problem because PTD-fused therapeutic molecules can deliver into brain tissues through the BBB. Of the various PTDs, PEP-1, consisting of three domains, has a greater efficiency when delivering target proteins, regardless of size, into cells. Additionally, PEP-1 has advantages in protein transduction, including high stability, a lack of toxicity, and a lack of sensitivity to serum [27]. Many reports have revealed that PTD fusion proteins can be used as novel delivery tools for various diseases, including neuronal diseases [27,28,29,30,31,32,33,34,35,36,37]. In the present study, a cell-permeable PEP-1–PIN1 fusion protein was constructed to investigate PIN1’s function in HT-22 hippocampal cells and a gerbil model of brain ischemic injury.

## 2. Materials and Methods

### 2.1. Construction and Purification of PEP-1–PIN1 Proteins

To facilitate the delivery of PIN1 into hippocampus HT-22 cells and into gerbil brain tissues, a PEP-1–PIN1 and a control PIN1 were constructed in the pET-15b vector by TA cloning, with or without PEP-1-expression vector. PEP-1–PIN1 and control PIN1 plasmid were transformed with *Escherichia coli* BL21 (DE3) cells and the cells were cultured and added to 0.5 mM IPTG (Duchefa, Haarlem, The Netherlands) at 37 °C for 4 h to express the PEP-1–PIN1 and control PIN1 proteins. The proteins were purified using a Ni^2+^-nitrilotriacetic acid Sepharose affinity column and PD-10 column chromatography and the concentration of purified proteins was confirmed using the Bradford assay [31,38].

### 2.2. Delivery of PEP-1–PIN1 into Hippocampal HT-22 Cells

Mouse hippocampal HT-22 cells were grown as described previously [31,32]. To confirm the delivered PEP-1–PIN1, various concentrations of PEP-1–PIN1 (0.5–5 μM) were added to HT-22 cells for 3 h, or were incubated with PEP-1–PIN1 (5 μM) for various times (30–180 min). In addition, the stable persistence of PEP-1–PIN1 in HT-22 cells was detected for 60 h after treatment with 5 μM protein. Next, the cells were harvested and delivered proteins were confirmed by Western blotting using polyhistidine antibody (Santa Cruz Biotechnology, Santa Cruz, CA, USA) as described previously [31,32]. Intracellular delivery of PEP-1–PIN1 into HT-22 cells was confirmed by visualization of the intracellular localization of polyhistidine 3 h after treatment with 5 μM protein.

### 2.3. Measurement of Oxidative Damage in HT-22 Cells

To assess the protective effects of PEP-1–PIN1 against the oxidative damage induced by the H_2_O_2_ in HT-22 cells, the cells were incubated with various concentrations of PEP-1–PIN1 (0.5–5 μM) for 3 h and exposed to H_2_O_2_ (1 mM) for 2 h. Thereafter, the MTT assay was performed to detect cell viability. The absorbance was measured at 570 nm using a Fluoroskan enzyme-linked immunosorbent assay (ELISA) microplate reader (Labsystems Multiskan MCC/340, Helsinki, Finland). Cell viability was expressed as a percentage of the normal control cells. [31,32,39].

To elucidate the effects of PEP-1–PIN1 on DNA fragmentation in HT-22 cells, the cells were incubated with PEP-1–PIN1 (5 μM) for 3 h and sequentially exposed to H_2_O_2_ (1 mM) for 6 h. To evaluate the production of ROS, HT-22 cells were incubated with PEP-1–PIN1 (5 μM) for 3 h and sequentially exposed to H_2_O_2_ (1 mM) for 1 h. Thereafter, TUNEL and 2′,7′-Dichlorofluorescein diacetate (DCF-DA) staining was performed. DCF-DA- and TUNEL-positive images were captured using a fluorescence microscope (Nikon Eclipse 80i, Tokyo, Japan) and the fluorescence intensity was measured using an ELISA plate reader (Fluoroskan Ascent, Labsystems Multiskan MCC/340, Helsinki, Finland) as described in previous reports [31,32,40].

### 2.4. Western Blot Analysis

Cell lysates containing equal amounts of protein volumes of sample buffer were separated in 12% SDS-PAGE and transferred to polyvinylidene difluoride membrane. Thereafter, the membrane was blocked with 5% nonfat dry milk for 1 h in a Tris-buffered saline (TBS) buffer containing 0.1% Tween 20. Membrane was then incubated with primary and appropriate secondary antibodies and immunoreactive protein bands were detected by enhanced chemiluminescence (ECL, Amersham Bioscience, Amersham, UK). The bands were quantified by Image J software 1.54h (NIH, Bethesda, MD, USA) as described previously [30,31,32].

### 2.5. Experimental Animals and Treatments

Male gerbils (65–75 g; 6 months old), obtained from the Experimental Animal Center, at Hallym University, were housed at a temperature of 23 °C, with humidity of 60%, and exposed to 12 h periods of light and dark with free access to food and water. All experimental procedures involving animals and their care conformed to the Guide for the Care and Use of Laboratory Animals of the National Veterinary Research and Quarantine Service of Korea. In addition, all animal experiments were performed according to the ARRIVE guideline (https://www.nc3rs.org.uk/arrive-guidelines, accessed on 26 July 2017) and were approved by the Hallym Medical Center Institutional Animal Care and Use Committee [Hallym 2019-51].

Ischemic injury was induced as previously described [30,32]. Briefly, the animals were anesthetized with a mixture of 2.5% isoflurane (Baxtor, Deerfield, IL, USA) in 33% oxygen and 67% nitrous oxide. Bilateral common carotid arteries were isolated and occluded using nontraumatic aneurysm clips. The complete interruption of blood flow was confirmed by observing the central retinal artery using an ophthalmoscope. After 5 min of occlusion, the aneurysm clips were removed from the common carotid arteries. The body temperature under free-regulating or normothermic (37 ± 0.5 °C) conditions was monitored with a rectal temperature probe (TR-100; Fine Science Tools, Foster City, CA, USA) and maintained using a thermometric blanket before, during, and after the surgery until the animals completely recovered from anesthesia. Thereafter, the animals were kept on the thermal incubator (Mirae Medical Industry, Seoul, South Korea) to maintain body temperature until the animals were euthanized. To examine the effect of PEP-1–PIN1 against ischemic injury, we divided the gerbils into five groups (*n* = 10 per group) and PEP-1–PIN1 (2 mg/kg), PIN1 (2 mg/kg), and PEP-1 (2 mg/kg) were intraperitoneally injected to gerbils 30 min after reperfusion.

### 2.6. Immunohistochemical Staining

For histological analysis, gerbils were sacrificed 7 days after ischemia/reperfusion and after brain tissues were extracted. Then, the brain tissues were cryoprotected, frozen, sectioned (50 μm), and immunohistochemical staining was performed as previously described [31,32,41]. Briefly, the sections from each animal were stained with a histidine antibody, neuronal nuclei (NeuN), Cresyl violet (CV), ionized calcium-binding adapter molecule 1 (Iba-1), glial fibrillary acidic protein GFAP (GFAP) and Fluoro-Jade B (FJB).

Personal computer (PC) images of the tissue were obtained using the CCD camera of an Axiophot light microscope (Carl Zeiss, Jena, Germany). The images of positive neurons were obtained by Apple scanner. The number of neurons was measured using an image analysis system equipped with a computer-based CCD camera (software: Optimas 6.5, CyberMetrics, Phoenix, AZ, USA). The staining intensity of the immunoreactive cells was evaluated as the relative optical density (ROD), which was calibrated as the % of control sham group as described previously [31,32,41].

### 2.7. Statistical Analysis

All statistical data were obtained using GraphPad Prism software (version 5.01; GraphPad Software Inc., San Diego, CA, USA). Values are shown as mean ± standard error of the mean from three experiments. Statistical comparisons between each group were performed using one-way analysis of variance with Bonferroni’s post-hoc test. Difference at *p* < 0.05 was considered statistically significant.

## 3. Results

### 3.1. Delivery of PEP-1–PIN1 into HT-22 Cells

**Figure 1:**
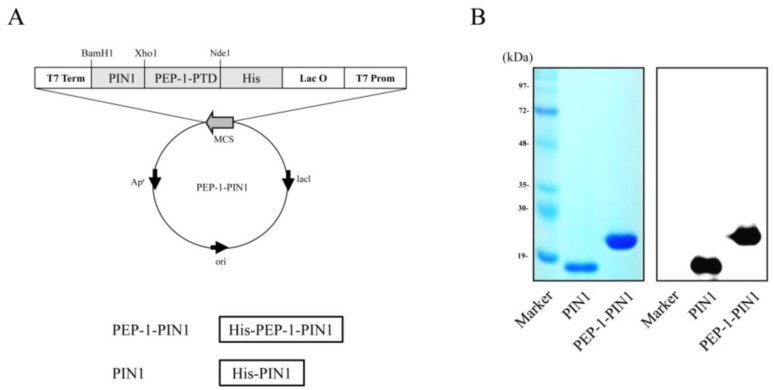
Construction of PEP-1–PIN1 and control PIN1 protein. Constructed map of PEP-1–PIN1 based on the pET-15b vector. PEP-1–PIN1 was designed to contain histidine, PEP-1-PTD and PIN1 (**A**). Purified PEP-1–PIN1 and control PIN1 were confirmed by Coomassie brilliant blue staining and Western blot analysis using anti-histidine antibody (**B**).

PEP-1–PIN1 expression vector was constructed using a pET-15b vector, which contained human PIN1 cDNA, six histidine, and PEP-1 peptide. A control PIN1 expression vector did not contain PEP-1 (Figure 1A). PEP-1–PIN1 and control PIN1 were overexpressed, purified, and identified by Western blotting (Figure 1B).

**Figure 2:**
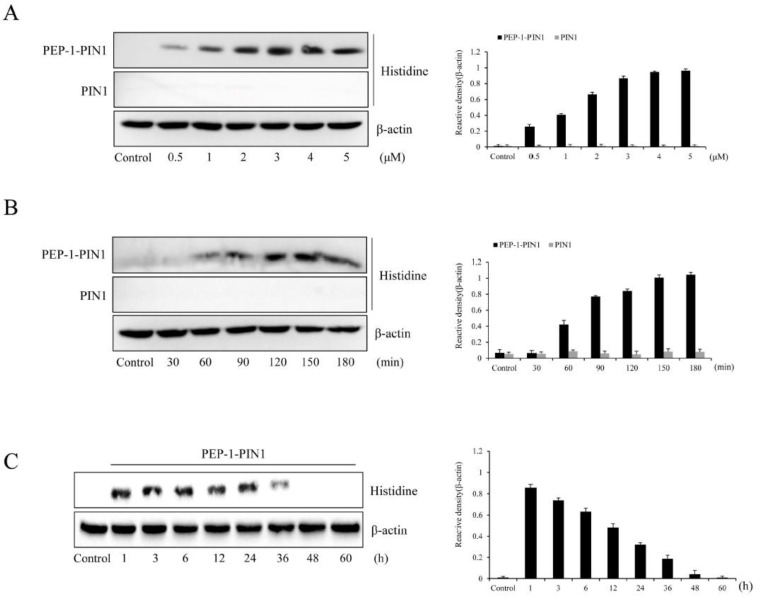
Delivery of PEP-1–PIN1 into HT-22 cells. HT-22 cells were treated with PEP-1–PIN1 (0.5–5 μM) for 3 h (**A**) or PEP-1–PIN1 (5 μM) for different time periods (30–180 min) (**B**). The intracellular stability of delivered PEP-1–PIN1 into the cells. HT-22 cells were treated with PEP-1–PIN1 for 3 h and washed. The cells were then further incubated for 1 to 60 h (**C**) and delivered PEP-1–PIN1 was assessed by Western blotting. The intensity of the bands was measured by a densitometer. Data are represented as mean ± SEM (n = 3).

Delivery efficacy of PEP-1–PIN1 was assessed by Western blot analysis after treatment with various concentrations (0.5–5 μM) of proteins or different incubation times (30–180 min). As shown in Figure 2A,B, PEP-1–PIN1 was delivered into HT-22 cells in a concentration- and time-dependent manner, while control PIN1-treated cells did not show delivery protein bands. Based on the results for PEP-1–PIN1 delivery into HT-22 cells, we set the concentration to be 5 μM and the incubation time to be 180 min.

**Figure 3:**
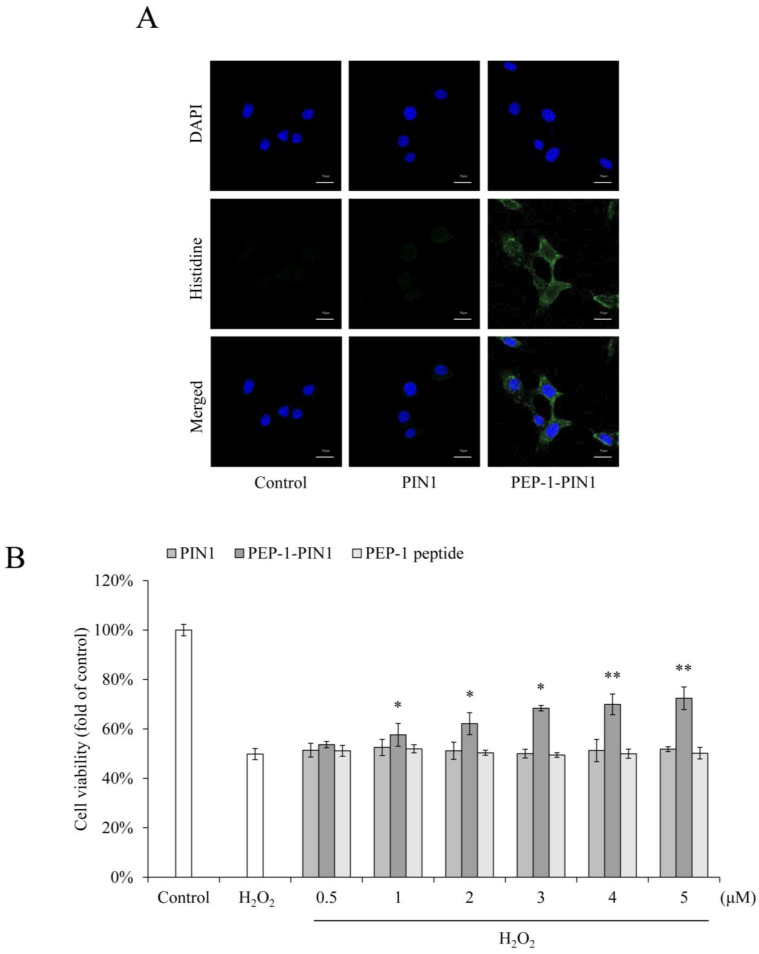
Effects of delivered PEP-1–PIN1 against H_2_O_2_-induced cell death. HT-22 cells were treated with PEP-1–PIN1 (5 μM) for 3 h. The localization of delivered PEP-1–PIN1 was confirmed by fluorescence microscopy (**A**). Scale bar = 20 μm. Effect of delivered PEP-1–PIN1 against H_2_O_2_-induced cell viability. The cells were pretreated with PEP-1–PIN1 (0.5–5 μM) for 3 h and exposed to H_2_O_2_ (1 mM) for 2 h. Cell viability was assessed by MTT assay (**B**). Data are represented as mean ± SEM (n = 3). * *p* < 0.05 and ** *p* < 0.01 compared with H_2_O_2_-treated cells.

We also determined the stability of PEP-1–PIN1 using Western blot analysis. HT-22 cells were treated with PEP-1–PIN1 for 3 h and washed. The cells were then further incubated for 1 to 60 h. Delivered PEP-1–PIN1 levels decreased over time and existed up to 36 h after protein treatment (Figure 2C). Intracellular distribution of delivered PEP-1–PIN1 was identified by immunofluorescence staining using histidine antibody (Figure 3A). In PEP-1–PIN1-treated HT-22 cells, histidine immunoreactive fluorescence was mainly shown in the cytoplasm of HT-22 cells. In contrast, control PIN1 treatment did not show histidine immunoreactive fluorescence in cells.

### 3.2. Effects of PEP-1–PIN1 on Oxidative Damage in HT-22 Cells

Oxidative stress was induced by incubation with H_2_O_2_. The protective effects of delivered PEP-1–PIN1 were then evaluated using MTT assay. As shown in Figure 3B, treatment with H_2_O_2_ significantly decreased cell viability to 50% of that of control cells. Cell viability was increased up to 72% in PEP-1–PIN1-treated cells, whereas control PIN1 and PEP-1 had no protective effect against H_2_O_2_-induced cell death.

**Figure 4:**
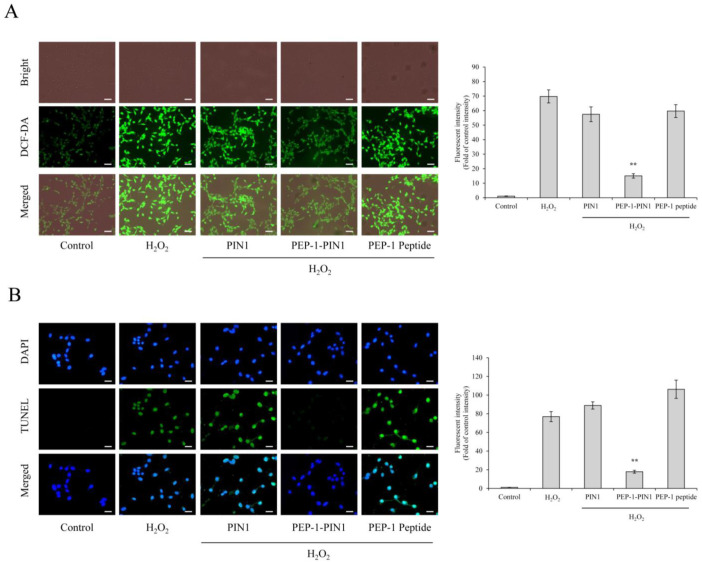
Effects of delivered PEP-1–PIN1 against H_2_O_2_-induced ROS production and DNA damage. HT-22 cells were treated with PEP-1–PIN1 (5 μM) for 3 h before treatment with 1 mM H_2_O_2_ for 1 h or 6 h. Intracellular ROS levels (**A**) and DNA damage (**B**) were determined by DCF-DA and TUNEL staining. Fluorescence intensity was quantified using an ELISA plate reader. Scale bar = 50 μm. Data are represented as mean ± SEM (n = 3). ** *p* < 0.01 compared with H_2_O_2_-treated cells.

We also performed DCF-DA and TUNEL staining to elucidate the effects of PEP-1–PIN1 on ROS production and DNA fragmentation. As shown in Figure 4, DCF and TUNEL fluorescence levels were very weak in control cells. Control PIN1 and PEP-1 peptide-treated cells showed DCF and TUNEL fluorescence levels similar to those of cells treated with H_2_O_2_ alone. In contrast, a strong DCF and TUNEL fluorescence levels were markedly decreased in cells treated with PEP-1–PIN1 than those in H_2_O_2_ alone and other treated cells.

### 3.3. Mechanisms Involved in the Protection of PEP-1–PIN1 against Oxidative Damage in HT-22 Cells

**Figure 5:**
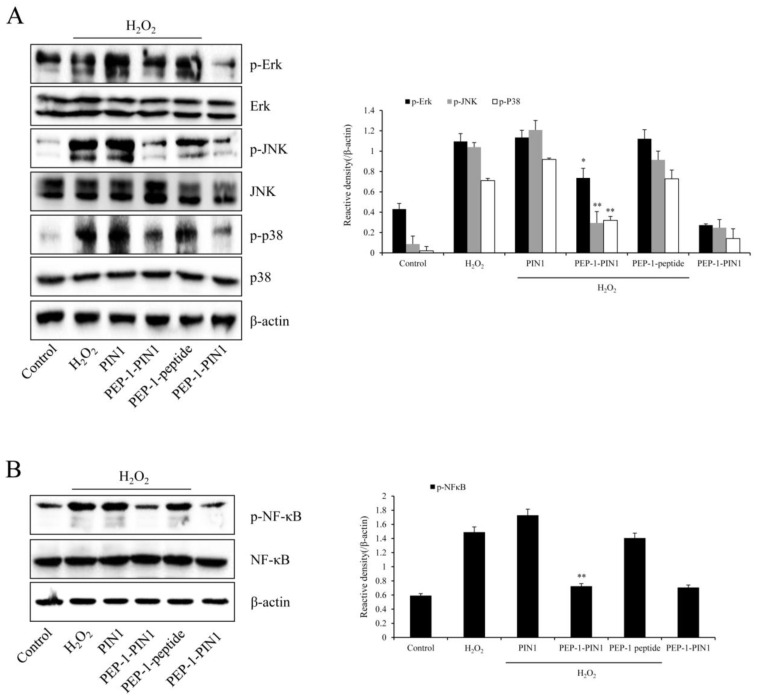
Effects of delivered PEP-1–PIN1 against H_2_O_2_-induced MAPK and NF-κB expression in HT-22 cells. The cells were treated with PEP-1–PIN1 (5 μM) for 3 h before being exposed to H_2_O_2_ (1 mM) for 60 min or 30 min, respectively. The expression levels of MAPKs (**A**) and NF-κB (**B**) were analyzed by Western blotting. The intensity of the bands was measured by a densitometer. Data are represented as mean ± SEM (n = 3). * *p* < 0.05 and ** *p* < 0.01 compared with H_2_O_2_ treated cells.

As the ERK, JNK, p38 (MAPK), and NF-κB signaling pathways are associated with oxidative stress-induced cell damage, we assessed expression levels of phosphorylated MAPKs and NF-κB in H_2_O_2_-exposed HT-22 cells using Western blotting (Figure 5). In cells treated with H_2_O_2_ only, phosphorylated MAPK and NF-κB expression levels were increased over those in control cells. In control PIN1 and PEP-1-treated cells, phosphorylated expression levels of MAPKs and NF-κB were similar to those in H_2_O_2_-treated cells. However, phosphorylated MAPK and NF-κB levels in PEP-1–PIN1-treated cells were significantly reduced over those in H_2_O_2_-treated cells.

**Figure 6:**
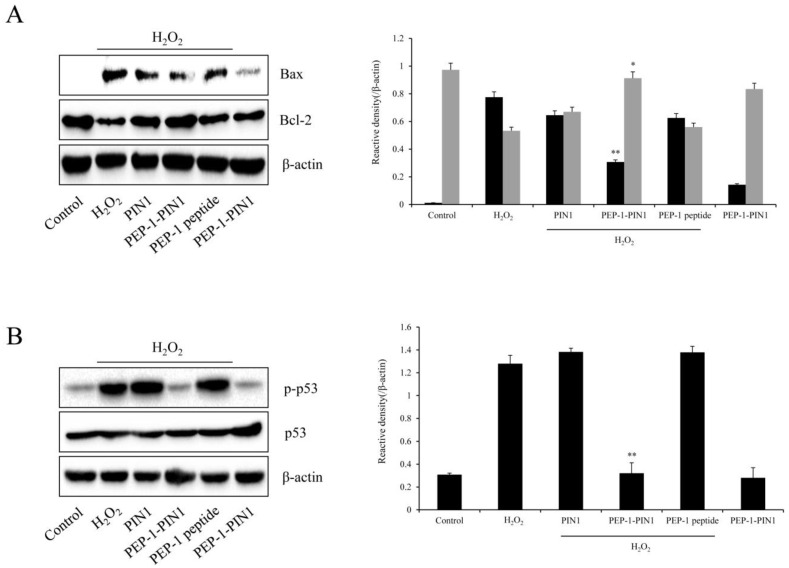
Effects of delivered PEP-1–PIN1 against H_2_O_2_-induced Bax, Bcl-2, and p53 protein expression in HT-22 cells. Three-hour pretreatment of HT-22 cells with PEP-1–PIN1 (5 μM) was followed by treatments with H_2_O_2_ (1 mM) for 120 min (Bcl-2), 240 min (Bax), and 360 min (p53). The expression levels of Bcl-2 and Bax (**A**) and p53 (**B**) were determined by Western blot analysis. The intensity of the bands was measured by a densitometer. Data are represented as mean ± SEM (n = 3). * *p* < 0.05 and ** *p* < 0.01 compared with H_2_O_2_ treated cells.

We also assessed expression levels of Bax, Bcl-2, and phosphorylated p53 in H_2_O_2_-exposed HT-22 cells using Western blotting (Figure 6). Bax and phosphorylated p53 levels were markedly increased, whereas Bcl-2 expression level was significantly reduced in H_2_O_2_-treated cells compared with those in control cells. In PIN1 and PEP-1-treated cells, expression levels of Bax, Bcl-2, and phosphorylated p53 were not significantly different from those in H_2_O_2_-treated cells. However, Bax, Bcl-2, and phosphorylated p53 expression levels in PEP-1–PIN1-treated cells were opposite those in H_2_O_2_-treated cells.

### 3.4. Protective Effects of PEP-1–PIN1 against Ischemic Injury in an Animal Model

**Figure 7:**
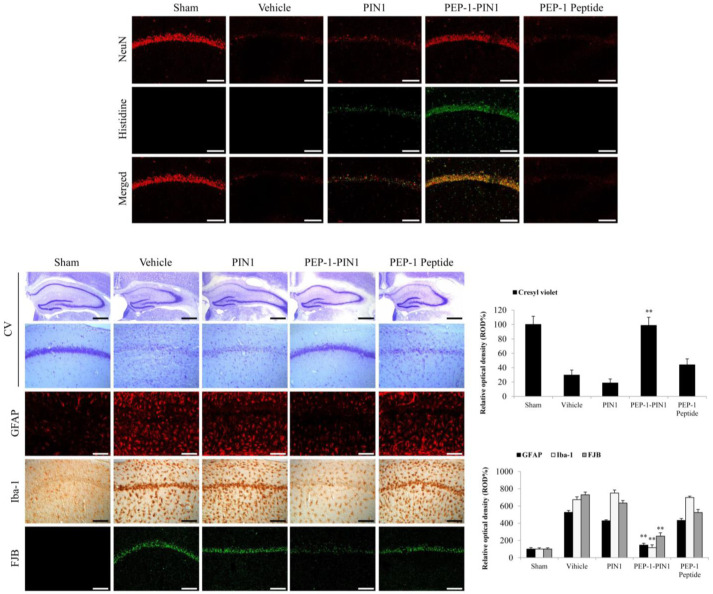
The neuroprotective effects of delivered PEP-1–PIN1 against ischemic damage. Gerbils were treated with a single injection of PEP-1–PIN1 (2 mg/kg) and sacrificed after 7 days. Delivery of PEP-1–PIN1 into the CA1 region of the hippocampus was determined by anti-histidine immunohistochemistry. Scale bar = 400 μm. The hippocampus was stained with NeuN, CV, GFAP, Iba-1 and FJB in sham-, vehicle-, PEP-1–PIN1-, PIN1-, and PEP-1-treated gerbils after ischemic injury. The graphic shows the relative numerical analyses of CV, GFAP, Iba-1 and FJB positive neurons in the CA1 region. Scale bar = 400 and 50 μm. Each bar represents the mean ± SEM of ten mice. ** *p* < 0.01, significant difference from the vehicle group.

The neuroprotective effects of PEP-1–PIN1 were confirmed by immunohistochemical staining in the CA1 region of the hippocampus at 7 days after ischemia (Figure 7). First, PEP-1–PIN1 and NeuN staining were performed to determine whether PEP-1–PIN1, when delivered into the brain, could protect neurons in the CA1 region. In sham-, PIN1-, and PEP-1-treated groups, histidine immunoreactivity was not shown in the CA1 region. However, histidine immunoreactivity was strongly detected in the PEP-1–PIN1-treated group.

NeuN- and CV-immunoreactive cells were detected throughout the CA1 region, whereas only a few NeuN-and CV-immunoreactive cells, because of neuronal cell death, were shown in the vehicle group. In the PEP-1-PIN-treated group, NeuN-and CV-immunoreactive cells were markedly increased compared with those seen in the vehicle group. In contrast, in PIN1- and PEP-1-treated groups, NeuN- and CV-immunoreactive cells did not show significant changes compared with those in the vehicle treated group. However, FJB stained cells showed the opposite pattern in the hippocampal CA1 region.

Furthermore, we determined whether PEP-1–PIN1 could protect activated astrocytes and microglia using GFAP- and Iba-1 immunoreactive staining. In the vehicle group, GFAP- and Iba-1-immunoreactivities were significantly increased compared with those in the vehicle group. In PIN1- and PEP-1 -treated groups, GFAP- and Iba-1-immunoreactivities showed a distribution pattern similar to those in the vehicle group. However, GFAP- and Iba-1-immunoreactivities were significantly decreased in the PEP-1–PIN1-treated group when compared with those in the vehicle group.

## 4. Discussion

PIN1, an 18 kDa protein, has a peptidyl-prolyl isomerase (PPIase) and a WW domain and plays a crucial role in different processes, including cell cycle, immune response, apoptosis, proliferation, and maintenance of the cytoskeleton [42,43]. It has been reported that PIN1 is distributed in the brain and has an important role in aging-related pathologies [44], an importance that has been demonstrated by the way that its expression decreases with aging in the myocardium [8,9,10]. Kuboki et al. have reported that PIN1 is required for NF-κB-DNA binding in hepatocytes during ischemia/reperfusion injury, suggesting that PIN1 is a critical regulator of NF-κB activation in hepatocytes and that it can protect against hepatic ischemia/reperfusion injury [45].

In this study, we investigated the roles of PIN1 after brain ischemic injury by using the cell-permeable PEP-1–PIN1 fusion protein and whether this protein protects against the cell damage in H_2_O_2_-induced HT-22 cells and in brain ischemic injury animal models. It is already known that many PTD-fusion proteins can be delivered into the cells by crossing the cell membrane [31,32,33,34,35,36,41,46]. We have observed that PEP-1–PIN1 fusion protein was delivered into HT-22 cells in a concentration- or time-dependent manner and that it degraded in a time-dependent manner.

To elucidate the effects of PIN1 on oxidative damage, we determined cell viability, ROS formation, and DNA fragmentation in H_2_O_2_-treated HT-22 cells after transduction of PEP-1–PIN1. This fusion protein markedly ameliorated H_2_O_2_-induced cell death by decreasing ROS formation and DNA fragmentation. Zhao et al. have reported that oxidative stress induced by H_2_O_2_ in HT-22 cells can increase ROS and cell death [47] and that oxidative stress induced by ROS also plays a key role in neuronal damage in the hippocampus [21,48,49].

As it is well known that ROS can modulate MAPKs and NF-κB signaling pathways, that MAPKs are known to play a crucial role in ROS-induced HT-22 cell death and that H_2_O_2_ can markedly increase the expression of MAPKs phosphorylation [48,50,51,52,53], we investigated the effect of PEP-1–PIN1 on changes in MAPKs and NF-κB pathways and observed that oxidative stress induced by H_2_O_2_ in HT-22 cells increased MAPKs and NF-κB phosphorylation, whereas PEP-1–PIN1 suppressed MAPKs and NF-κB phosphorylation. It is also known that excessive ROS can lead to mitochondria damage and the release of pro-apoptotic proteins such as Bax [54,55]. Therefore, we determined protein levels of Bcl-2 and Bax. HT-22 cells after treatment with H_2_O_2_ showed a significant increase of Bax levels but decrease of Bcl-2 levels. However, PEP-1–PIN1 modulated the expression levels of these proteins, suggesting that PEP-1–PIN1 might play a crucial role in cell survival against oxidative stress.

To elucidate the effects of PEP-1–PIN1 on brain ischemic injury, we investigated the morphology of the hippocampus using immunohistochemistry in a brain ischemic injury model. We found that PEP-1–PIN1, when delivered into the CA1 region of the hippocampus and crossing through the BBB, protected against cell death in the brain. It has been reported that the activation of astrocytes and microglia is associated with ischemic brain injury, and that this has been used as a marker for the detection of ischemic brain injury [56,57,58,59]. Several studies have reported that activation of astrocytes and microglia can induce ischemic injury and change the morphology in the CA1 region of the hippocampus [60,61,62,63]. Consistent with these results, we also observed the suppression of the activation of astrocytes and microglia after treatment of PEP-1–PIN1.

In this study, we showed the protective effects of PEP-1–PIN1 against brain ischemic injury, but further studies are needed, including confirmation of the role of PEP-1–PIN1 in signaling pathways in primary hippocampal neurons and in ischemia animal models. Additionally, studies on the exact function of PEP-1–PIN1 in brain ischemia are required. Although further study for the exact molecular mechanisms still needs to be explained, we expect PEP-1–PIN1 to help develop an efficient therapeutic agent for brain ischemia.

## 5. Conclusions

We showed that transduced PEP-1–PIN1 into HT-22 cells could attenuate cell death by not only reducing intracellular ROS levels, but also modulating MAPK and apoptosis signaling. Although further studies are needed to elucidate its protective mechanisms, our results demonstrate that PIN1 plays a neuroprotective role against ischemic brain injury, suggesting that it might be used as therapeutic protein for ischemic brain injury.

## Data Availability

The original contributions presented in the study are included in the article, further inquiries can be directed to the corresponding authors.
